# The Underlying Process of Prosocial Behavior Among Soldiers: A Terror Management Theory Perspective

**DOI:** 10.3389/fpsyg.2021.770723

**Published:** 2022-01-07

**Authors:** Ido Heller, Samer Halabi

**Affiliations:** Academic College of Tel Aviv-Yaffo, Tel Aviv-Yafo, Israel

**Keywords:** intergroup relations, prosocial behavior, soldiers, terror management theory, threat

## Abstract

The mortality salience (MS) hypothesis postulates that anxiety elicited by mortality awareness leads people to develop negative emotions toward those who hold values inconsistent with their worldview faith. We explored this hypothesis in a sample of 76 Israeli combat soldiers, who were asked to reflect on either their mortality or dental pain. Subsequently, participants reported their motivation to help a father in need who was either an Arab (outgroup) or a Jewish Israeli (ingroup), as well as their perceptions of threat by Arab Israelis. Regression analysis indicated that mortality reminders intensified soldiers’ perception of threat by the outgroup, leading to an increased desire to assist a Jewish-Israeli father, and a decreased motivation to help an Arab-Israeli one. The findings demonstrate the pronounced effects of MS on soldiers involved in frequent combat actions in terms of evoking negative emotions leading to reluctance to help unarmed civilian outgroup members. Recommendations for soldiers’ pre-deployment psychoeducation sessions are provided.

## Introduction

Manifestations of human altruism and willingness to assist others are evident in most human societies. Scholars have been studying human behavior in an attempt to understand the psychological processes that lead people to behave kindly, rather than cruelly, toward others (Farsides et al., 2013). Extensive research on prosocial attitudes has shown that people’s motivation to assist others is broad and includes not only personality factors such as agreeableness and altruism (Graziano et al., 2007), but also factors related to power disparities between benefactor and recipient and their groups’ social status (Dovidio et al., 2006; Nadler and Halabi, 2006). The present research considered additional factors that could influence individuals’ prosocial motivation. In particular, based on terror management theory (TMT) (Greenberg et al., 1986; Solomon et al., 1991) and social identity theory (Tajfel and Turner, 1979), we tested the relationship between mortality salience (MS; i.e., increased awareness of one’s mortality) and prosocial behavior among Israeli soldiers serving in the West Bank, while additionally considering the mediating role of perceived threat (Stephan and Stephan, 2000).

### Terror Management Among Soldiers

Military conflicts may intensify existing rivalry and negative attitudes between social groups (Dechesne et al., 2007; Van den Berg et al., 2009; Mitchell, 2015). Experiencing threat to self and group can reinforce the distinction between “us” and “them” and lead to more stereotyping processes (Tajfel and Turner, 1986). Consistently, and relevant to the present research context, a recent study found that Dutch soldiers serving in Afghanistan identified more with their ingroup values, and reported more stereotyping behaviors against outgroup individuals to maintain a more positive ingroup identity (Van den Berg and Soeters, 2009; Van den Berg et al., 2009). Such circumstances of intense threat may change perceptions of the outgroup, usually for the worse (Fritsche et al., 2009).

Note that military operations tend to perpetuate the threat of death, increasing mortality salience (MS) levels and exacerbating the hostility between combat soldiers and outgroup members (Van den Berg et al., 2009). The MS effect on soldiers’ attitudes is so powerful, in fact, that Dutch and Turkish soldiers’ willingness for international cooperation in Afghanistan was much lower in the front line compared to safer locations (Dechesne et al., 2007). Interestingly, while Dutch and Turkish soldiers operated together as part of NATO forces, concerns with death caused many of them to exclude other soldiers of a different national and religious background despite their shared political goals, and they reported greater need for closure and more national identification (Dechesne et al., 2007).

Terror management theory (TMT) is a social psychology paradigm that provides in-depth insight on existential threat, mortality awareness, and intergroup relationships. Consistent with TMT, reminders of death were found to affect participants to favor people who belong to their groups and to derogate others critical of their country (e.g., Greenberg et al., 1990). Moreover, and more relevant to the current study context, MS was shown to lead to physical distancing from foreigners (Ochsmann and Reichelt, 1994) and to greater sensitivity to national symbols (Dechesne and Kruglanski, 2004), which may be more profound in the case of soldiers.

### The Effects of Mortality Salience on Intergroup Relations

TMT proposes that individuals’ awareness of death has a significant effect on their attitudes and appreciation of values (Greenberg et al., 1986; Solomon et al., 1991). The MS hypothesis suggests that once individuals are confronted with an existential terror, they seek to establish their worldview faith (e.g., nationality, political affiliation, religion) and self-esteem, which are considered two major anxiety-buffering mechanisms (Pyszczynski et al., 1999).

Once an individual’s worldview faith is challenged, negative emotions toward anyone who may pose a threat to the worldview faith are increased. Rosenblatt et al. (1989) were the first to test this hypothesis. They found that mortality reminders increased the punishments that American court judges set to prostitutes, demonstrated the negative effect of MS on attitudes toward people who represent a different perceived set of values. More recently, Burke et al. (2010) reviewed 164 studies that examined terror management techniques and showed that MS exacerbated negative emotions, derogation, and stereotyping toward outgroup members. More specifically, it was shown that people who were reminded of death and dying were particularly inclined to defend stereotypic images of the outgroup which were part of their cultural worldviews (Schimel et al., 1999). In similar vein, Hobfoll et al. (2006) found that intensified exposure to terrorism during the early 2000s increased Jewish Israelis’ support for ethnic exclusion, authoritarianism and political violence against Palestinian-Arabs both in the Occupied Territories and within Israel.

### Death Awareness and Prosocial Behaviors

In addition to the findings reviewed above, the literature has also emphasized the effects of MS on prosocial behaviors. Since the early 70s, scholars have explored prosocial behaviors in diverse aspects such as the bystander effect (Latane and Darley, 1970), the severity of the recipient’s emotional distress (Shotland and Huston, 1979), and, more recently, the types of assistance to the outgroup, such as searching for a mother’s lost daughter (Cuddy et al., 2007), or donating money and clothes (Hirschberger et al., 2008). Furthermore, recent research has provided evidence for an MS effect on motivation for prosocial behaviors. For example, following 9/11, Steinberg and Rooney (2005) found that Americans’ awareness of their mortality intensified their prosocial behavior as they became more involved in rescue and charitable activities. Similarly, Jonas et al. (2002) reported that when interviewed in front of a funeral home (thereby reminded of mortality) people showed greater willingness to donate money to their favorite charity than when interviewed three blocks away. Importantly, this study further showed that in the MS condition, participants donated more money to a charitable cause, but only to an ingroup (American) cause, not an international one. Likewise, Schimel et al. (2006, Study 1) found that Canadian participants were most likely to forgive a hockey player for committing an act of instrumental aggression when the player belonged to their hometown team, but not when the offender was an outgroup member. Consistently, Hirschberger et al. (2008) found that Israeli students were more motivated to assist their favorite charity after being prompted to reflect on their mortality.

However, limited studies focused on MS and prosocial behaviors among military personnel, and on threat perception that may affect this relationship. This research is particularly noteworthy because it tests prosociality under extreme conditions. Can compassion be extended to an adversary who is not merely an outgroup member, but one who might also pose a direct threat? Extensive research has established the relationship between MS and increased prosocial behavior (Hirschberger, 2015), yet research has also pointed to the dynamic role of existential concerns in the MS-prosocial behavior relationship.

Indeed, research building on the intergroup threat theory (ITT; Stephan and Stephan, 1985, 2000; Stephan et al., 2008), has indicated that threat emanates from experiencing a challenge to one’s goals and well-being. Reactions toward such threats are predominantly negative. Threat, especially on the intergroup level, was found to be highly related to negative behaviors toward its source that range from aggression, hostility and discrimination to warfare and other forms of open conflict (Stephan et al., 2008, 2015). Consistently, the current research tests MS’s effect on perceptions of Jewish Israeli soldiers toward the outgroup, Arabs, and their willingness to offer assistance to a needy Jew or a needy Arab, while assessing the mediating role of perceptions of Arabs as threat. The conflict in the region presents a complex reality of an ongoing clash between combat soldiers and civilians (Jews and Arabs, respectively). This situation involves cognitive and mental hardships, as it tends to lead soldiers to develop stereotypes about and suspicions against the entire population considered an enemy (Ben-Ari, 1998; Van den Berg et al., 2009). Given the unique environments in which combat soldiers operate, it is imperative to understand the psychological and social dynamics that play a role in prosocial behavior as a terror mitigation strategy.

### The Present Study

It may be expected that as individuals’ motivation to assist outgroup members is often lower than their desire to help ingroup members (Cuddy et al., 2007) MS will exacerbate this trend. MS negatively affects one’s attitudes toward an outgroup member who holds a dissimilar worldview faith (Solomon et al., 1991), and reduces the desire to assist an outgroup member in general (Levine et al., 2005). Particularly, in the present study, integrating concepts from social identity theory and TMT, we explore the willingness of Jewish Israeli soldiers to assist a needy father who is either an Israeli Jew or an Israeli Arab. Further, we investigate how the perceived threat by outgroup members mediates soldiers’ motivation for prosocial behavior.

To expand the existing literature on the MS-prosocial behavior relationship, we introduce two hypotheses. First, we hypothesize an interaction effect of MS and ethnicity. Following a reflection on mortality, Jewish Israeli soldiers would be more motivated to assist an ingroup member, a Jew, than an outgroup member, an Arab. Second, based on studies that examined the MS effect on hostility and threat in general (e.g., Pyszczynski et al., 2006) and among NATO soldiers in Afghanistan in particular (Soeters et al., 2007; Van den Berg et al., 2009), we hypothesize that the perceived outgroup threat would be intensified in the MS condition and become a critical motivator of prosocial behavior toward ingroup members.

## Materials and Methods

### Participants

The participants were 76 Jewish Israel Defense Forces (IDF) soldiers (57 men) aged 21–31 (*M* = 24.1, *SD* = 1.8). All had extensive military experience in the front line (*M* = 2.13 years, *SD* = 0.97). Participants completed their mandatory service (3 years for men, two for women) in combat units (“Activity Group A+” according to the IDF’s definition: in that group, soldiers serve in hostile areas and engage in dangerous military operations (Israel Defense Forces Human Resource Division, 2013). Thus, at the time of study, they were serving in the reserves. Participants were randomly assigned to one of four conditions within a 2 (MS vs. Control) × 2 (Jewish vs. Arab recipient) between-subjects design. As recommended by Cohen (1988) and Giner-Sorolla (2020), we performed *post hoc* sensitivity power analysis using G*Power v. 3.1.9.2 (Faul et al., 2009), which indicated that the current sample size was sufficient to detect a large effect size (*f* = 0.376) (Cohen, 1988) with power = 0.90 for α = 0.05.

### Materials and Procedure

The effect of MS on individuals’ attitudes, behaviors, and emotions may be reliably significant when no other factor primes participants with MS. If such a factor does exist, it may be a confounding variable that compromises the research findings’ internal validity (Hirschberger et al., 2002; Taubman-Ben-Ari and Findler, 2003, 2006). Thus, we portrayed the study as an assessment of attitudes toward various social groups in Israel. Potential participants were recruited via social media and emails were sent to college students describing it and asking them to partake in a brief survey on age, gender, ethnicity, marital status, and military service background. Next, and without knowing the study’s real purpose, those who identified themselves as active combat reservists were invited by email to take part in the actual study. The experimenters sent 101 online questionnaires to candidates, of whom 25 did not reply or neglected to complete the survey (response ratio: 75%).

After introducing the study to the participants, several general questions concerning their gender, age, marital status, military rank, and years of military service (in hostile zones) were introduced. Following these background questions, the MS manipulation was introduced: half (*n* = 38) of the reservists were asked to briefly answer two questions: (1) What will happen to you when you physically die? and (2) What emotions does the thought of death arouse in you? This approach was first used in Rosenblatt et al. (1989). In the control condition (*n* = 38), participants were asked similar questions, but the words “dental pain” were used instead of death-related words, based on the procedure employed by Greenberg et al. (1992).

Following the MS manipulation, a word search puzzle that included 14 words was introduced (Hirschberger et al., 2002). None of these words (e.g., “apple,” “fall,” and “squirrel”) ware associated with death or negative thoughts. In agreement with previous TMT studies (Solomon et al., 1991; Hirschberger et al., 2002), this filler was used to maximize the effectiveness of the MS manipulation by allowing the participants several minutes to adhere to their worldview faith (Pyszczynski et al., 1999).

Next, the ethnicity of the person in potential need was manipulated. Participants read a fictionalized news report based on Cuddy et al.’s (2007) scenario. Our story described the harsh consequences of a natural disaster and reported a father who had lost his son in a hiking trip due to a flood in Israel’s Negev desert. As in Cuddy et al. (2007) and Halabi et al. (2019), we manipulated the father’s ethnicity by using different first names and background descriptions. Half the participants (*n* = 38) read about a Jewish Israeli father called Ben, from Herzliya, a predominantly Jewish city in central Israel. The other half (*n* = 38) read about an Arab Israeli father called Muhammad, from Kafr Kana, an Arab town in northern Israel. According to the Israel Central Bureau of Statistics (2018), more than 487 Jewish boys born in 2016 were named Ben and one in every five Muslim boys born in 2016 was named Mohammed.

The ethnicity manipulation was evaluated in a pilot study. Mirroring the procedure used in the main study, 48 Jewish Israeli participants were presented with an identical news report that described a Jewish Israeli (*n* = 22) or an Arab Israeli father (*n* = 26) who needed help. After reading the report, among other questions, participants were asked to indicate the father’s mother tongue as an indirect indication of his ethnicity. As expected, participants who read the report with the Jewish name correctly indicated that the father’s mother tongue was Hebrew, and those who read the report with the Arab name correctly indicated that the fathers’ mother tongue was Arabic, χ^2^(1, *N* = 48) = 40.28, *p* < 0.001.

As suggested, the report described how the father lost his young son in a flood. The father needed more people to help in the search operation and to rest at someone’s home for the night. Next, among other questions that included irrelevant items to obscure the true purpose of the study, we assessed the participants’ perceptions of Arabs as a threat. The threat was measured using both indirect and direct items. For the indirect threat measure, participants were asked to estimate the ratio of several social groups in Israel’s general population (e.g., Jewish settlers in the West Bank), including Arab Israelis, on a 100-point Likert scale, from 0 = “no representation in the general population” to 100 = “full representation” (Hjerm, 2007). In a previous study, subjective perceptions of outgroup size were associated with perceptions of threatened ingroup interests, which in turn were related to negative outgroup attitudes (Schlueter and Scheepers, 2010). Specifically, Smooha (2004, 2012) found that 70.1% of Israeli Jews in 2003, and 51.5% in 2012, were threatened by the growing of the Arab population in Israel.

For the direct threat measure, participants were asked to report on a 7-point Likert scale, from “low” to “extreme,” to what extent Arab Israelis were considered threatening by Jews. This direct measure, involving an assessment of the risks of harm to one’s physical wellbeing (or realistic threat; Stephan et al., 2008, 2015), was based on a measure of threat extensively used to explore relations between Jews and Arabs in Israel (Smooha, 2012, 2016, 2019).

Following the measure of threat, participants were asked to rate on a 7-point Likert scale (from 1 = “not at all” to 7 = “very much”) the extent of their endorsement of two statements regarding the news report: (a) Are you willing to assist Ben/Muhamad in searching for his son? (b) Are you willing to host Muhamad/Ben for one night in your home? Ratings for the two items (*r* = 0.891) were averaged to obtain a single measure of willingness to help the needy father, with higher scores reflecting greater willingness to provide assistance. After completing the questionnaires, all participants were debriefed about the true purpose of the study.

## Results

In our main analyses, we examined the effects of the two experimental conditions, Mortality Salience (MS vs. control) and Ethnicity of the potential help recipient (Arab vs. Jewish) and their interaction with willingness to offer help and perceptions of threat.

### Willingness to Offer Help

**FIGURE 1:**
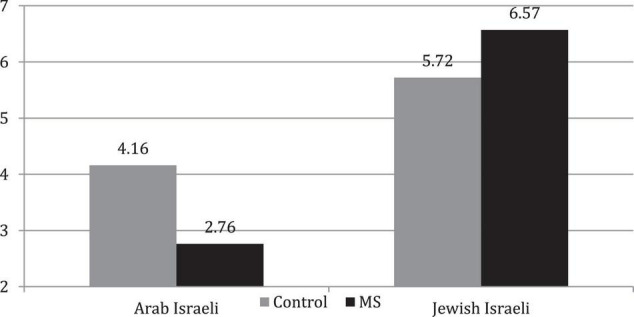
Participants’ willingness to help a Jewish Israeli vs. Arab Israeli father.

A 2 (MS vs. Control) × 2 (Jewish vs. Arab recipient) ANOVA performed on participants’ score on the willingness to help the needy father, revealed a significant main effect for ethnicity, *F*_(1, 76)_ = 351.60, *p* < 0.001, η^2^ = 0.830, and a main effect of MS manipulation, *F*_(1, 76)_ = 3.67, *p* = 0.059. However, the ANOVA revealed a 2-way interaction, *F*_(1, 76)_ = 62.04, *p* < 0.001, η^2^ = 0.463. The interaction was due to the finding that, compared to the control condition, MS reduced participants’ willingness to assist the Arab Israeli father, *M*s: 4.16 (*SD* = 0.66), and 2.76 (*SD* = 0.71), respectively, *t*(72) = 6.83, *p* < 0.001, *d* = 2.03, while it increased their willingness to assist the Jewish father, *M*s: 5.72 (*SD* = 0.65), and 6.57 (*SD* = 0.67), respectively, *t*(72) = 4.27, *p* < 0.001, *d* = 1.507 (see Figure 1).

### Perceived Threat

**FIGURE 2:**
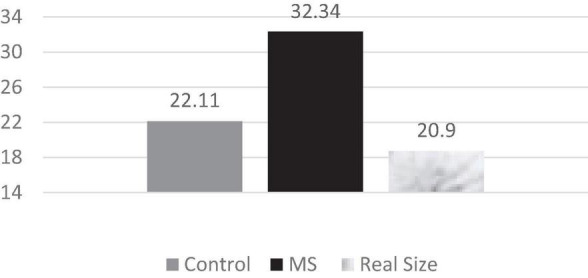
Participants’ estimation of the percentage of Arabs in the general Israeli population compared to the actual percentage (Israel Central Bureau of Statistics, 2018).

A one-way ANOVA was performed on the direct (i.e., perception of Arabs as threatening) and indirect (i.e., estimation of the ratio of Arabs in the general population) measures of threat. As predicted, there was a statistically significant difference in threat perception between participants in the MS condition who tended to perceive Arabs as more threatening than participants in the control condition, *M*s: 6.12 (*SD* = 0.83) and 4.03 (*SD* = 1.38), respectively, *F*_(2, 74)_ = 7.89, *p* < 0.001, η^2^ = 0.434. Moreover, MS was found to significantly affect soldiers’ estimation of the ratio of Arabs in Israel’s population, *F*_(2, 74)_ = 8.99, *p* < 0.001, η^2^ = 0.509, In particular, participants in the MS condition estimated that ratio as significantly higher than in the control condition (32.34 and 22.11%, respectively); the precise ratio of Arabs in Israel at the time of study was 20.9% (see Figure 2).

**FIGURE 3:**
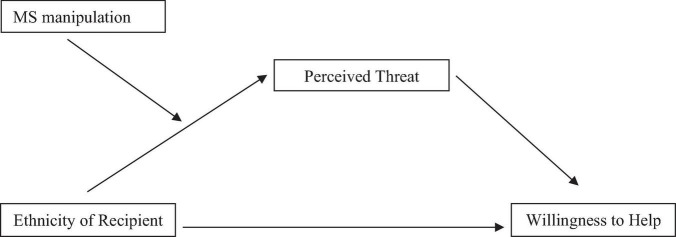
The study’s moderation-mediation model.

To further examine our predictions, we conducted a moderated mediation analysis using Hayes’s (2013) PROCESS macro (Model 7). The conceptual framework of the proposed model is shown in Figure 3. In this model, ethnicity of potential needy was entered as the independent variable, MS manipulation as the moderator, perception of Arabs as threat as the mediator, and willingness to assist the Arab Israeli father as the dependent variable. In line with our predictions, the conditional indirect effect of ethnicity on reduced willingness to assist the Israeli Arab father through higher perception of Arabs as threat was significant for participants in the MS manipulation condition, β = -0.14 (*SE* = 0.09), CI_95_ = [–0.416, –0.026], but not for those in the control condition, β = −0.07 (*SE* = 0.102), CI_95_ = [−0.295, 0.115]. That is, among those in the MS manipulation condition—but not among those in the control condition—the path from ethnicity to reduced willingness to help the father was mediated by increased perception of Arabs as a threat.^1^

## Discussion

The mortality salience (MS) hypothesis asserts that mortality reminders lead people to protect themselves against death concerns by adhering to their worldview faith and engaging in congruent actions (Rosenblatt et al., 1989). Previous studies revealed that MS is an important factor in explaining prosocial behavior (Jonas et al., 2002; Schimel et al., 2006; Hirschberger et al., 2008). The present research expands previous terror management findings to military personnel and reveals an additional component, perception of threat, which causes prosocial tendencies to decrease under conditions of MS. In accordance with previous studies (Hirschberger et al., 2010), it shows that MS leads people to become self-protective altruists. Jewish Israeli soldiers reported higher motivation for benevolent acts toward an ingroup member, yet they were less likely to do the same for an outgroup member. This phenomenon occurred mostly because Arab Israelis not only differ from Jews in terms of their ethnicity, but also, if not mainly, because they are perceived as hostile to the predominant Jewish Israeli worldview faith (Hobfoll et al., 2006). By displaying ingroup favoritism in helping a stranger in need, the soldiers were able to maintain psychological equilibrium in the face of perceived existential threat.

### The Relationships of Mortality Salience, Perceived Threat, and Prosocial Behavior

Our findings indicate that MS exacerbated soldiers’ perception of threat by those with a dissimilar worldview faith. According to group threat theory (Hjerm, 2007), prejudice and intergroup hostility are reactions to the *perception* of threat, rather than the actual threat that the ingroup faces. Considering that relations between Israeli Arabs and Jews are very complex (e.g., Bar-Tal and Sharvit, 2008; Smooha, 2012, 2019), we found that threat constituted a very important element in in explaining the connection between MS and prosocial behavior among Jewish soldiers.

Like many if not most Jewish Israelis, IDF soldiers seem to fear physical attacks by Arab Israelis deemed to hold a worldview faith hostile to their dominant ideology (Johnson, 2010). Our findings supported the group threat theory’s hypothesis in the military context. Soldiers in the MS condition reported higher levels of threat by Arab Israelis compared to soldiers in the control condition. Importantly, the MS effect on perceived threat was confirmed not only by the direct self-report question, but also by an indirect question that revealed disproportional higher estimates of the Arab Israelis population by participants in the MS condition compared to the control condition and to its actual size, by 73 and 46%, respectively. By using two different questions—self-report of threat (direct) and estimation of minority group size (indirect/vague)—we examined both emotional-behavioral and cognitive-abstract perceived threat.

Previous studies on the influence of threat in the military context have mainly focused on the adverse effects of fear, such as combat stress reactions (Solomon et al., 1988), the relation between threat and morality (Shamir et al., 2000), and fear of current and future dangers in hostile zones (Van den Berg and Soeters, 2009). However, they neglected to focus on the perceived threat by collective groups of civilians. Our study addressed this gap in the literature. Another gap addressed by the current research is that previous studies focused on the death threat and international collaboration (Dechesne et al., 2007; Van den Berg and Soeters, 2009), but not on prosocial behavior. Our findings clearly indicate that perceived threat by a social group played a crucial factor in the motivation to help a stranger.

Hence, MS that contributes to the perception of threat may impair soldiers’ altruistic values and behaviors toward outgroup members, while at the same time reinforce benevolent behaviors toward other members of the ingroup, even if they are strangers. Note that the current findings show how MS affects IDF soldiers in terms of helping an outgroup member involved in an incident, who is supposed to elicit empathy and caring that are unrelated to ingroup conduct. Previous research did indicate that MS reduced prosocial behavior toward the rival group only when the ingroup could be held accountable for the incident; when the ingroup was not held responsible, MS motivated more prosocial acts (study conducted in the Israeli-Arab context; Hirschberger and Ein-Dor, 2011). Thus, consistent with a self-protective altruism perspective that ingroup responsibility toward an adversarial group is threatening when death is salient because it undermines the protection provided by the belief that one’s group is morally superior, MS primed with threat, decreases prosocial acts toward a member of an adversarial group (Hirschberger and Pyszczynski, 2011).

### Limitations

Several methodological limitations should be addressed. First, our research used a fictionalized story, and, though floods in the Negev desert happen frequently in Israel, future studies should examine the motivational and behavioral manifestation of the disruption of terror management processes under more realistic settings and among soldiers during combat deployment.

Second, Israeli soldiers perceive the military as the emblem of patriotism, an emotion intensified by serving in a combat unit. Thus, other variables that were not measured, such as patriotism, could increase the soldiers’ motivation to help the ingroup more than the outgroup (Sasson-Levy, 2008).

Third, based on Halabi et al. (2008), reinforcing existing power relations between advantaged (Jewish-Israeli soldiers) and disadvantaged groups (Arab-Israelis) could be an additional factor affecting the benefactors’ prosocial behavior motivation to maintain their group’s social status. Recent studies on helping processes have shown that advantaged group members often try to maintain their group’s positive social identity and advantaged position by adopting prosocial behaviors toward the disadvantaged group, behaviors that reflect a motivation to maintain the social inequality between the groups (Nadler et al., 2009; Bruner et al., 2015; Nadler and Halabi, 2015). In addition, we acknowledge that although participants were very conscientious in their response in the present study (no missing data and no outliers in the measures), we did not assess actual helping responses but relied on the participants’ self-reports. While some have argued that such measures are not always necessary when predicted results are obtained (Sigall and Mills, 1998), including such additional measures in future research and investigating actual helping behaviors would be most valuable in future research as these measures could affirm that participants faithfully followed the instructions and that measures of this type could further illuminate the underlying dynamics. Especially in times of change, advantaged groups are strongly motivated to preserve their higher status (Sidanius and Pratto, 1999; Saguy and Dovidio, 2013). These motivations may be manifested in subtle, seemingly benevolent forms of bias (Pratto and Walker, 2001), such as helping, which may or may not be consciously strategic.

Finally, though the nature of the Arab-Israeli conflict and its intensity may be well evident within the current unique and hard-to-get sample of male and female combat soldiers with extensive military experience, we acknowledge that because of practical constraints in the recruitment of participants, we used relatively small samples, which may have limited statistical power. However, the effect sizes we observed were generally sufficient, and power analysis revealed that level the of statistical power (0.57) was within the norm of social psychology research and even exceeded the mean power of 0.35 (Bakker et al., 2012), and was sufficiently sensitive to reliably detect medium effect sizes (Cohen, 1988). However, to further establish the research arguments, future research could explore a larger sample with more equal distribution of female and male combat soldiers, what in turn may enable a closer consideration of the role of gender, MS, threat and prosocial preferences.

## Conclusion and Recommendations

The current research provides a significant contribution in exploring the underlying dynamics of prosocial ambivalence of combat soldiers under MS conditions. Our findings showed that terror management processes might underlie the decision to engage in or refrain from prosocial activity. On the one hand, soldiers may choose to assist others if it is consistent with their worldview. On the other hand, they may refrain from prosocial acts due to a symbolic threat to their worldview faith and/or a perception of actual threat.

MS is inherent to military service far more than in the general population, and terror management processes invariably affect soldiers’ psychological equilibrium, as they are in constant life-threatening situations. They affect their mental health (Delahaij et al., 2006), acceptance of the risk of dying (Van den Berg and Soeters, 2009), motivation for military service (Taubman-Ben-Ari and Findler, 2006) and, as our study shows, also prosocial values. Future studies may test the relationship between MS and perceived threat and intergroup relations among the general population and among other populations that are expected to serve the community while facing mortality threats, such as police officers. Scholars should further explore prosocial behaviors, intergroup relations and MS using various types of assistance that may provoke threat, such as providing dependency- or autonomy-oriented assistance, such as providing the outgroup member with the tools and knowledge to solve a problem (Halabi et al., 2008), that may potentially promote the recipient’s social status.

Finally, military psychologists need to be aware that worldview faiths strengthen soldiers’ ingroup bias and reinforce perceptions of threat by outgroups. Accordingly, they should recognize that military forces recruit soldiers from diverse ethnic, religion, and political backgrounds. Hence, they should emphasize the importance of productive group dynamics, mutual goals, and human values, so that under MS conditions, soldiers will not perceive civilian outgroup members as outright enemies.

## Data Availability Statement

The raw data supporting the conclusions of this article will be made available by the authors, without undue reservation.

## Ethics Statement

The studies involving human participants were reviewed and approved by the Academic College of Tel Aviv-Yaffo Ethics Committee. The patients/participants provided their written informed consent to participate in this study.

## Author Contributions

IH conducted the research, was responsible for data collection, analyzing data, and took the lead in writing the report. SH was involved in designing the research and writing the report. Both authors contributed to the article and approved the submitted version.

## Conflict of Interest

The authors declare that the research was conducted in the absence of any commercial or financial relationships that could be construed as a potential conflict of interest.

## Publisher’s Note

All claims expressed in this article are solely those of the authors and do not necessarily represent those of their affiliated organizations, or those of the publisher, the editors and the reviewers. Any product that may be evaluated in this article, or claim that may be made by its manufacturer, is not guaranteed or endorsed by the publisher.

## Footnotes

1 We also tested an alternative model (Hayes, 2013; Model 8) in which MS manipulation was entered as the independent variable, ethnicity of potential needy as the moderator, perception of Arabs as threat as the mediator, and willingness to assist the Arab Israeli father as the dependent variable. In line with previous findings demonstrating that MS leads people to help more in general (e.g., Jonas et al., 2002; Steinberg and Rooney, 2005; Hirschberger et al., 2008) results indicated that there was a significant direct and indirect path from the MS manipulation x Ethnicity of Needy interaction toward willingness to help the father through increased perception of Arabs as a threat (i.e., zero was not included in the 95% confidence interval which were –3.14 to –1.77, indicating that the indirect effect significantly differed from zero).
